# The influence of an educational intervention on nursing students’ domestic violence knowledge and attitudes: a pre and post intervention study

**DOI:** 10.1186/s12912-022-00884-4

**Published:** 2022-05-07

**Authors:** Frances Doran, Thea van de Mortel

**Affiliations:** 1 School of Health and Human Sciences, Southern Cross University, PO Box 150, Lismore, NSW 2480 Australia; 2 School of Nursing and Midwifery, Griffith University, Parklands Drive, Southport, QLD 4222 Australia

**Keywords:** Domestic violence, Intimate partner violence, Student nurses, Education

## Abstract

**Background:**

Nurses, as the largest group of health professionals, have a key role in recognising, mitigating and preventing domestic violence. However, studies demonstrating effective undergraduate educational interventions are lacking. The research aim was to compare undergraduate nursing students’ knowledge and attitudes about domestic violence before and after an educational intervention on domestic violence and explore their views on the most useful teaching strategies.

**Methods:**

A quasi-experimental pre and post design was used to determine the impact of an educational intervention. Australian nursing students enrolled in a first-year undergraduate subject were invited to participate. The educational intervention included a 40-min pre-recorded lecture on domestic violence, and a two-hour face-to-face workshop facilitated by an expert, supported by readings. Students completed a pre- and post-intervention online anonymous survey using a validated instrument, the Inventory on Beliefs and Attitudes towards Domestic Violence. Wilcoxon signed rank tests were used to compare pre and post intervention results.

**Results:**

Approximately 400 students completed the voluntary workshop; 198 students completed the pre survey, 176 completed the post survey and 59 (13.1%) completed both. Post intervention, participants indicated stronger agreement on 15 of 22 items. The inventory score became significantly more positive (Z = -3.196, *p* = .001, CI -.206—-0.067) post intervention. Of the 173 students who indicated post intervention which forms of education they found useful, 38.2% considered face-to-face tutorials to be the most useful education modality.

**Conclusions:**

This study demonstrates the effectiveness of even a small educational intervention in changing attitudes, and creating awareness and knowledge of the context, prevalence, perpetrators, and significant associated burden of illness related to domestic violence, and nurses’ responsibility to support victims.

## Background

Internationally, domestic violence (DV), also called intimate partner violence, is violence that occurs between current or former intimate partners [1, 2], and is recognised as a priority women’s health issue in Australia [3] and globally [1]. Domestic violence can include physical, sexual, emotional, cultural/spiritual and financial violence, and a wide range of controlling, coercive and intimidating behaviours [4]. The term DV was adopted in this study as it is consistent with terminology used in the Australian National Community Survey towards Violence Against Women [4].

Domestic violence occurs in all settings and among all socioeconomic, religious and cultural groups, and genders [3]. Indigenous, young and pregnant women, those separating from their partners, with a disability, or experiencing financial hardship, women from culturally and linguistically diverse backgrounds and people identifying with diverse sexual orientation are at a higher risk [3]. Approximately 17% of women have experienced DV since the age of 15 compared to 6.1% of men [3]. Women are twice as likely to be killed by an intimate partner than men [3]. Domestic violence against women and children markedly increased during COVID-19 [5], creating a ‘pandemic within a pandemic’ [6]. Globally, the ‘lock-down’ measures negatively impacted vulnerable groups, and home became an even less safe space [7–9].

Domestic violence is associated with significant negative health, economic, social, and emotional consequences, contributing to 5.1% of the burden of disease for Australian women aged 15–44 and 11% for indigenous women [10]. In 2016–2017, 4,600 Australian women and 1,700 men were admitted to hospital due to DV [2]. Children who witness DV have a high risk of mental health issues and behavioural and learning difficulties [1]. Given the associated morbidity, those affected by DV are high users of health services [2].

Victims who receive an initial positive response to DV disclosure are more likely to seek further help to escape violence [11]. Yet an extensive review of health system responses suggests that interventions to reduce the DV-related harm are lacking [12]. Nurses have an important role in screening for victims and facilitating access to assistance [13]. However, barriers to effective prevention and care include stereotypical and gendered attitudes that normalise violence within intimate partner relationships and victim-blame, coupled with insufficient preparation in university undergraduate courses on gendered violence, and screening, communication and management strategies to deal with it [12, 14]. Studies exploring barriers to DV screening suggest the need for increased training to enhance appropriate clinical responses [15–17].

Despite global support for the integration of DV content into undergraduate health professional degrees [3, 18], approximately 80% of health professionals have never received DV management training [19]. While nursing students who receive some training about DV are better prepared for clinical practice with an understanding of the context of DV compared to those who have not had such training [20], research reveals integrated DV assessment, intervention content and planned clinical experiences within nursing undergraduate degrees are lacking as are rigorous evaluations of effective DV educational strategies in nursing education [12, 14, 20–25]. This translates to a lack confidence to respond appropriately to DV [21].

In Australia, less than 1% of nursing subject outlines specifically refer to DV [24], and undergraduate nursing and midwifery students report less than three hours DV content in their degree [26]. Similarly, while many nursing students understand the nature and consequences of DV, others display stereotypical and gendered attitudes that normalise violence within intimate partner relationships [27]. A key component of undergraduate education is learning to address attitudes and myths [4] and build clinical competence in identifying DV indicators and responding appropriately to disclosures. The Queensland DV Taskforce suggests undergraduate courses provide opportunities to change attitudes about DV and equip graduates with the tools to understand and address it [28].

Several studies have reported on specific DV educational interventions in undergraduate health professional curricula. A meta-analysis on DV digital education for pre and post-registration health professionals indicated improved outcomes compared to no intervention or traditional teaching methods, although the level of evidence was poor [19]. Only one study had registered nurse participants and the intervention focussed on detecting child abuse rather than the broader spectrum of DV. Maquibar et al. [29] exploration of the impacts of DV educational content in a Spanish undergraduate nursing program found participants self-reported increased knowledge and confidence in identifying cases post training. However, there were no pre and post comparisons of knowledge or attitudes, and no details were provided on the format or duration of training, limiting the utility of the findings.

Several studies have examined DV training delivered via simulation, however, most examined self-reported knowledge or attitudes post training [30, 31] with no pre-intervention assessment to make a comparison against. Blumling et al. [32], however, provided a DV lecture and standardised patient (SP) simulation depicting a DV victim to American nursing students, finding statistically significant increases in confidence pre to post-lecture and post-simulation, and in knowledge about DV pre to post-lecture but not post-simulation.

This review demonstrates a lack of DV education in undergraduate nursing programs coupled with limited evaluation of the influence of specific education strategies on nursing students’ attitudes towards, and knowledge of DV. Our study aimed to evaluate the influence of an educational intervention on nursing students’ DV knowledge and attitudes and ascertain student perceptions on the most useful DV teaching strategies.

## Methods

### Design, intervention and participants

A quasi-experimental pre- and post-intervention design was used to explore the impact of an educational workshop on undergraduate nursing students’ knowledge of, and attitudes towards, DV. The educational intervention included a 40-min pre-recorded lecture on DV, and a two-hour face-to-face workshop facilitated by a DV expert, supported by readings, and covered behaviours included in DV; its health impacts, prevalence, and contributing factors; the role of the nurse and importance of screening, and the use of appropriate language with DV victims. Four hundred and fifty-two nursing students enrolled in a first-year undergraduate subject were invited to participate by the Primary Investigator via an online announcement on their subject website where they were directed to a link to the pre- and post-workshop anonymous online surveys hosted on Qualtrics. Data were collected between August and October, 2019.

### Ethics approval

The research was approved by the Southern Cross University Human Research Ethics Committee (ECN-17–009). Students were provided with information about the study, including ethical considerations and what participation would involve. Participation was voluntary and the survey was anonymous. Survey completion was considered to indicate consent.

### Instrument

The Inventory on Beliefs and Attitudes towards DV, a validated tool [33], was used to explore students’ DV knowledge and attitudes. The 18-item inventory explores perceptions of behaviours that constitute DV, students’ personal attitudes towards DV, knowledge of triggers of DV, and abuser background and behaviour of the abused, and the nurse’s role on a 5-point Likert scale where 1 = strongly agree and 5 = strongly disagree. The published inventory was slightly modified to include one item on the perceived prevalence of DV and three on the impact of DV on children. The survey elicited demographic information on age, gender and cultural background. Students were asked to indicate which forms of teaching delivery they found most useful and had the opportunity to provide written comments. Both surveys took less than fifteen minutes to complete.

### Data Analysis

A student generated anonymous code was used to match pre- and post-intervention responses. Within-subject pre- and post-intervention statistical analysis involved self-reported ratings in response to statements about DV. Data were analysed in SPSS 25 [34]. Differences across time for items were analysed using Wilcoxon signed-rank tests for non-parametric data, as the data were ordinal, and non-normally distributed. Scale reliability was determined via Cronbach’s alpha.

## Results

The same facilitator delivered 15 workshops across three campuses to approximately 400 nursing students (~ 200 from one campus and 100 each from the two smaller campuses). One hundred and ninety-eight (198) students completed the pre-intervention survey, 176 completed the post-intervention survey and 59 (13.1%) completed both. Attendance was not compulsory. On average 26 nursing students attended each workshop. The majority were female (86.4%), aged 18–29 years (67.8%) and non-indigenous (93.2%) (Table 1).

**Table 1:** Demographic characteristics of participants

Variable	Percentage (n)
**Gender**	
Female	86.4% (51)
Male	13.6% (8)
**Age (years)**	
18–29	69.5% (41)
30–39	25.4% (15)
40–49	5.1% (3)
**Identity**	
Indigenous Australian	3.4% (2)
Other Australian	93.2% (55)
Not specified	3.4% (2)

### Comparison of pre-intervention to post-intervention scores

Cronbach’s alpha was 0.882 on both pre and post versions indicating good reliability. After the intervention, participants indicated stronger agreement on 15 of 22 items. The inventory score mean overall became significantly more positive (Z = -3.196, *p* = 0.001, CI -0.206—-0.067) post intervention. Items that were statistically more positive are noted in Table 2. Gender did not significantly influence the change in scale scores.

**Table 2:** Inventory on Beliefs and Attitudes towards Domestic Violence: change in nursing students’ scores post intervention

	**Statement**	** + /–**	**n**	**z**	***p*(2-tailed)**
**Forms of DV**					
1	If a partner yells abuse this is a form of DV	+	59	-0.43	.667
2	If a partner repeatedly criticises the other one to make them feel bad or useless, this is a form of DV	+	59	.000	1.000
3	If a partner forces the other partner to have sex this is a form of DV	+	59	-0.05	.958
4	If a partner tries to control the other by denying them money, this is a form of DV	+	59	-1.93	.054
**Nursing and DV**					
5	Nurses and midwives have an important role in providing emotional and practical support to women who experience DV	+	59	-0.46	.647
6	Nurses and midwives have an important role in detecting DV through risk assessment and screening	+	59	-0.43	.670
7	Education on DV is an important part of the undergraduate nursing and midwifery curriculum	–	59	-0.12	.904
**Myths about women’s role in DV**					
8	Domestic violence is a private matter to be handled in the family	+	59	-0.82	.415
9	If a woman gets hit by her partner, she probably deserves it	+	58	-0.11	.912
10	Women who perpetrate DV often do it in self-defence or retaliation for previous violence	+	59	-2.76	.006*
11	It is hard to understand why women stay in violent relationships	–	59	-1.16	.245
**Characteristics of abusers and abused**					
12	Most DV abusers are from lower income groups	–	59	-0.39	.697
13	Most women who are abused are from low-income groups	–	59	-0.35	.727
14	The majority of DV abusers are men	+	58	-3.48	< .001*
**Power and responsibility**					
15	One of the causes of DV is seen as the power imbalance	+	57	-1.97	.048*
16	Domestic violence can be excused if it results from people getting so angry they temporarily lose control…	–	58	-0.24	.809
17	In some circumstances using physical force in intimate relationships is acceptable…	–	58	-0.33	.740
18	Domestic violence is never excusable	–	58	-0.31	.755
**DV and Children**					
19	Witnessing DV harms children's development	+	59	-0.63	.532
20	Exposure to DV is a form of child abuse	+	59	-2.19	.029*
21	Children living in a DV household experience emotional and psychological trauma	+	58	-0.47	.637
22	Violence against women is common in our community	+	59	-3.61	< .001*

^*^p < .05

### Most useful forms of DV education delivery

Nursing students (*n* = 173) indicated post intervention which forms of DV education they found most useful (Table 3).

**Table 3:** Forms of delivery most useful to learn about domestic violence

Type of delivery	N (%)
Face to face tutorial	66 (38.2)
Face to face lecture	44 (25.4)
Clinical placement	6 (3.5)
Clinical laboratory	4 (2.3)
Online materials	3 (1.7)
Self-directed reading	2 (1.2)
Other	48 (27.7)
Total	173

‘Other’ responses included 34 detailed examples; most suggested a combination of strategies or more interactive strategies including simulations, speakers with personal experience of DV, case studies, role plays and opportunities to talk with victims of DV.

## Discussion

This study highlights the value of a brief DV education program incorporating a pre-recorded lecture, supporting readings and a two-hour workshop delivered by a content expert. Nursing students demonstrated a significant, mildly positive improvement in knowledge of, and attitudes towards DV. Ison [35] indicates that educational preparation of students is strengthened when delivered by experts who can make explicit the gendered nature of DV in both personal and professional spheres. Students’ understanding of the behaviours that constitute DV improved post-intervention, in keeping with broader Australian studies that note that more people are recognising non-physical behaviours as DV [4]. The 2017 National Community Attitudes towards Violence against Women survey findings indicate that most Australians have an accurate knowledge of what constitutes violence against women and do not endorse it [4].

A significant underlying factor in DV is inequality between men and women and the lesser status of women compared with men in society [1]. These factors contribute to the gendered drivers of violence [36], which are expressed through stereotyped constructions of masculinity, disrespect for women and beliefs blaming women for the violence. Our students did not display these negative attitudes and gender differences were not apparent in this study, despite Australian men demonstrating a higher acceptance of violence towards women [4]. This may be because the proportion of males in our sample was small. A Turkish study examining the attitudes of 750 midwifery and nursing students towards violence against women found students’ ability to recognise violence was inversely related to traditional attitudes towards women [37]. Maquibar et al. [29] also found myths about DV persisted among nursing students in Spain and they lacked understanding of the complexity of DV, which impedes accurate identification of cases and subsequent care. Domestic violence education at foundation level must encompass an exploration of myths and attitudes that need to be addressed prior to entering clinical practice [26]. Findings of a scoping review that explored health care provided to those experiencing DV within primary health care settings in America, the United Kingdom, Sweden and Brazil, identified lack of educational preparation as an impediment to comprehensive care and an urgent need for nurses to be better educationally prepared [38].

Although nursing students’ attitudes and understanding of DV shifted positively after the intervention, the results were weakly positive indicating other options need to be examined. Students commented positively on the lecture, the workshop, the content addressed and the use of videos and discussion exercises within class and offered suggestions for more interactive and experiential strategies to deliver DV education. Similar calls for more interactive and ‘hands-on’ DV education including role plays, simulations, mentored learning in the clinical setting and learning about screening from previous victims of DV have been suggested in several studies [25, 39]. Several studies have demonstrated significant improvements in DV knowledge, understanding, communication self-efficacy and/or confidence to assist DV victims following DV simulations in concert with other strategies such as scripts, videos and presentations in third year Spanish nursing students [5] and fourth year medical students in Mozambique [40].

Several studies have demonstrated improved attitudes and skills following longer teaching sessions, although there has been no comparison of outcomes between shorter and longer DV education sessions. For example, significant shifts in developing more positive attitudes towards DV and gender roles were noted with first-year undergraduate midwifery students [41] following participation in a gender equality course that included 2-h interactive sessions for 10 weeks. Similarly, Turkish student nurses and midwives exposed to a 2-h lecture for 14 weeks on DV, displayed a significant reduction in traditional attitudes group and improved their ability to recognise the signs of violence and their views on their professional roles in addressing violence compared to a control group [42]. Although feedback was not sought on the teaching method, the results demonstrate the potential benefits of nursing curricula inclusive of the DV content.

Statistically significant improvements in knowledge about and/or attitudes towards clients experiencing DV following educational interventions on DV have been noted with registered nurses across a range of international settings. These have included a mixture of didactic lectures, role plays, and case reports over seven days (Turkey) [43], didactic and simulation activities (America) [32, 44] a 3-h interactive session (Saudi Arabia) [45], and two 3-h sessions (Sweden) [46]. Several authors recommended continuous training rather than one-off interventions are these appear more valuable in influencing practice [46, 47]. The latter suggest that integrated DV content should be mandatory as those who were exposed to it were better able to better recognise the signs of violence, and traditional attitudes about women decreased compared to a control group [47].

Violence against women is a global public health problem of epidemic proportions, requiring urgent action from the health sector to integrate issues related to violence into clinical training [18]. To be able to respond appropriately, at the very minimum, nurses must understand the relationship between exposure to violence and women’s ill health [18]. Nurses need comprehensive undergraduate education on gender-based violence to prepare a workforce that has positive attitudes, is committed, and equipped for their important clinical role to identify and respond to DV. Negative attitudes, misinformation and lack of commitment must be addressed in undergraduate education [37]. Otherwise, those who access care might be dismissed, doubted, and blamed for causing the violence, and, consequently, might not only feel hopeless but miss out on life-changing support [45]. This study was undertaken before the COVID-19 pandemic, but given the increase in those experiencing DV since COVID-19 it is even more important for nurses to be better educationally prepared with a comprehensive focus on DV within curricula to ensure nurses are well equipped to respond appropriately [38].

Nursing students are best supported to do this in safe environments within their undergraduate teaching so they can practice their skills and develop confidence to effectively intervene in real peoples’ lives once they graduate and enter the health workforce. This is fundamental given the high rates of access to health care services by women who experience DV. Nurses are the biggest professional group of the health workforce and are the most preferred health professional women feel comfortable making a disclosure of DV to [14].

### Limitations

Study limitations included the low numbers responding to both surveys and implementation at one university, which limit the ability to generalise the results. Even on anonymous online surveys, participants may also display socially desirable responding [48], which may influence the results to some extent. On the positive side a validated instrument was used to collect these data and the scale reliability was better in this study than in the original instrument validation study (Cronbach’s alpha (0.882 vs 0.646) [33].

## Conclusions

This study demonstrates the effectiveness of a small DV educational intervention in changing attitudes, and creating awareness and knowledge of the context, prevalence, perpetrators, significant associated burden of illness of DV, and nurses’ responsibility to support victims. Nurses need to feel comfortable to ask questions to identify DV and support women and children with interventions to reduce the burden of illness and save lives. Further research may identify the best combination of educational practices at undergraduate level to achieve this outcome.

## Data Availability

The dataset analysed during the current study is available from the corresponding author on reasonable request.

## Abbreviations

AIHW: Australian Institute of Health and Welfare
DV: Domestic violence
SPSS: Statistical Package for Social Sciences
